# An Intelligent Infrastructure as a Foundation for Modern Science

**Published:** 2025-08-12

**Authors:** Satrajit Ghosh

**Affiliations:** McGovern Institute for Brain Research, Massachusetts Institute of Technology, Cambridge, MA; Department of Otolaryngology - Head and Neck Surgery, Harvard Medical School, Boston, MA

## Abstract

Infrastructure shapes societies and scientific discovery. Traditional scientific infrastructure, often static and fragmented, leads to issues like data silos, lack of interoperability and reproducibility, and unsustainable short-lived solutions. Our current technical inability and social reticence to connect and coordinate scientific research and engineering leads to inefficiencies and impedes progress. With AI technologies changing how we interact with the world around us, there is an opportunity to transform scientific processes.

Neuroscience’s exponential growth of multimodal and multiscale data, and urgent clinical relevance demand an infrastructure itself learns, coordinates, and improves. Using neuroscience as a stress test, this perspective argues for a paradigm shift: infrastructure must evolve into a dynamic, AI-aligned ecosystem to accelerate science. Building on several existing principles for data, collective benefit, and digital repositories, I recommend operational guidelines for implementing them to create this dynamic ecosystem, aiming to foster a decentralized, self-learning, and self-correcting system where humans and AI can collaborate seamlessly. Addressing the chronic underfunding of scientific infrastructure, acknowledging diverse contributions beyond publications, and coordinating global efforts are critical steps for this transformation.

By prioritizing an intelligent infrastructure as a central scientific instrument for knowledge generation, we can overcome current limitations, accelerate discovery, ensure reproducibility and ethical practices, and ultimately translate neuroscientific understanding into tangible societal benefits, setting a blueprint for other scientific domains.

Infrastructure transforms societies^1^. It powers cities, connects communities, and fuels the engines of progress. From power grids and highways to satellites and the internet, it moves people, goods, information, and ideas. It also serves as the backbone of scientific advancement^2,3^. Particle accelerators, genome sequencers, and AI systems are examples of infrastructure that open new frontiers of knowledge, allowing us to peer into the origins of the universe, decode the building blocks of life, and transform our everyday lives. But, as environmental, energy, and health challenges grow more complex and urgent, we will need to transform the world’s science and engineering infrastructure to solve them.

To this day, scientific infrastructure is usually seen as a static scaffolding that is built for specific purposes. This results in infrastructure that is often fragmented, overly specific, and short-lived. Typically built by individual labs, such systems suffer from variable quality and limited scalability. Even larger scale, governmentally supported infrastructures are often non-interoperable, compartmentalized, and dependent on funding cycles. With some exceptions, they have mostly operated without coordination, without community input, and without a vision for growth. This stifles scientific and technological progress, and especially at the cutting edge, where innovation is rapid, infrastructure lags behind.

What we need is an infrastructure that is intelligent (that can learn, collaborate, decide, and generate) and designed for the dynamic nature of the scientific enterprise. It must be an integral and integrating part of an active ecosystem for science. As such it is no longer sufficient for infrastructure to merely adapt to the changing landscape. Instead, it should *shape* it, and be placed at the very center of a vision where knowledge is not just captured but created through intelligent systems that can handle evolving complexity of scientific endeavors. AI is increasingly well positioned to serve this role.

In this perspective, I examine the evolving relationship between science and AI through my own lens of neuroscience. Neuroscience has a significant potential for societal impact and is an ideal testbed for a new discovery science paradigm. I assess the gaps in current neuroscience infrastructure that hinder progress, and recommend a set of operational guidelines for a new infrastructural paradigm – a scientific ecosystem that changes how we work together to solve our greatest challenges.

## Neuroscience and AI: A Synergistic Playground

**Figure 1.**
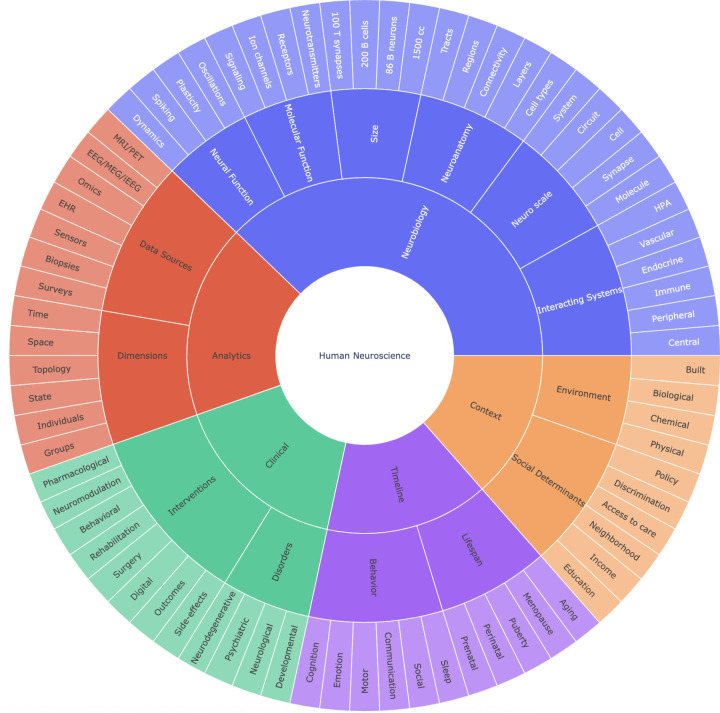
Complexity of Human Neuroscience. To fully connect knowledge in human neuroscience research, our system will have to assemble data and information across all these elements and connect to the fundamental neuroscience work being conducted in other species. Such a system needs to contextualize any piece of information by its provenance and meaning, which will require rich data and metadata to be available. Further, curating, processing, and connecting such information will require a usable and programmable interface that allows both humans and machines to read and write information. To ensure privacy, such transactions will need to be authorized appropriately depending on the sensitivity of the information. Only an intelligent system in partnership with experts who understand the nuances and failure modes of research can connect this large information space and make it accessible appropriately.

Neuroscience research presents enormous challenges. Each human brain contains about 86 billion neurons and roughly the same number of non-neuronal cells^4^, and over a 100 trillion connections, so understanding neuroanatomy, molecular dynamics, and neurophysiology across spatial and temporal scales in multiple environments is a monumental task. In turn these inform the study of brain development, behavior, as well as interventions for learning disabilities, neurological conditions, and neuropsychiatric disorders. All this requires observing individuals across a broad range of biological, environmental, developmental, and lifespan dimensions (see Figure 1). Because many of these studies are extremely difficult or currently impossible to perform in humans, much of our current understanding is derived from studies in non-human species.

In terms of infrastructure, these inquiries have led to significant advancements in tools and technologies and have spurred the launch of numerous brain initiatives around the world. These initiatives have aimed at delivering a blueprint for brain function by fostering collaboration, standardizing protocols, and supporting scalable data production and integration. Several large-scale consortial projects have been funded by the US NIH BRAIN Initiative^5^ to map cells (BICAN) and circuits (CONNECTS) to behavior (BBQS) and are emblematic of this new era. These consortia are generating comprehensive datasets across species. They required a level of coordination and data integration that needed the creation of an entire neuroinformatics project portfolio and the development of new standards, computational tools, and several data repositories^6^.

These are not the only projects. Efforts like BigBrain^7^ and the Fly brain^8^ project are mapping the cellular anatomy of various organisms with increasing resolution, and EBrains^9^, an EU effort to accelerate collaborative brain research. Initiatives such as ABCD^10^, ABIDE^11^, OpenNeuro^12^, HCP (Human Connectome Project^13^), ENIGMA^14^, and ADNI (Alzheimer’s Disease Neuroimaging Initiative^15^) have accelerated large-scale human neuroimaging and genetics data collection and sharing within and across nations and labs. Large-scale registries and biobanks, such as the Swedish National Patient Register^16,17^ and UK Biobank^18^, are increasingly complementing these studies, enabling epidemiological research to be conducted at an unprecedented scale by integrating genomic, imaging, and extensive clinical data from vast populations.

Problematically, these projects leverage technologies from the last decade, and the backbone infrastructures supporting them suffer from fragmentation, limited coordination, and a limited roadmap for sustainability, and are often isolated efforts. As examples, there are multiple ongoing and uncoordinated efforts across the ecosystem to develop neuroscience schemas and terminologies, atlases, workflow systems, models, and more infrastructure. There are significant redundancies across these efforts. All of these efforts could benefit from a connected ecosystem, where access to expertise, knowledge, and ongoing efforts are quickly available, and coordinated to synergize alongside AI advances.

AI holds immense potential to take on this staggering volume and complexity and revolutionize neuroscience by connecting information, transforming computation, and generating new knowledge^19,20^. Over the last decade, the evolution of AI technologies has been rapid, driven by advancements in computational power, data availability, and algorithmic innovation, many inspired by neuroscience. Today’s AI landscape is characterized by foundational models, large-scale pre-trained models that can be adapted for a wide range of downstream tasks, offering many generalization capabilities. Reinforcement learning has enabled AI systems to improve representations through trial and error, a process that mirrors aspects of scientific discovery and human learning. Furthermore, “agentic paradigms” are gaining traction, where AI systems are designed to act autonomously, make decisions, and interact with their environments, moving beyond passive analysis to active experimentation. Reasoning capabilities are increasingly being integrated into AI, allowing systems to infer, deduce, and generate hypotheses. Embodied intelligence, where AI systems can perceive and interact with the physical world, also holds immense promise for robotics and experimental control in neuroscience^21^.

For all that, AI in and for neuroscience is still in its infancy. Most foundational models for neuroscience^22–26^ and similar efforts to understand the brain through AI models^27–29^ are highly constrained. For AI to effectively assist in reshaping this field, three conditions must be met. First, comprehensive and reliable information from labs, protocols, data, and publications need to become fully available, similar to the plethora of text that training of large language models (LLMs) rely on. The information needs to span both successes and failures and allow for a comprehensive understanding of the entire data lineage, from pre-acquisition through all processing stages. Second, this rich information needs robust and specialized infrastructure that can support the massive data volumes, facilitate seamless integration of diverse data types, and provide computational resources for the training and deployment of sophisticated AI models. Moreover, it needs to enable rapid cycles of experimentation, data analysis, and model refinement. Third, these interactions within the neuroscientific ecosystem need to produce information and knowledge that is continuously assessed for validity, disseminated broadly, and are treated as communication signals in an AI-based system.

This nexus between neuroscience and AI is a synergistic playground. The goal is to move towards a future where AI not only assists but actively participates in the scientific process, designing and even running experiments, interpreting results, and proposing subsequent steps, ultimately driving a more agile and efficient path to neuroscientific understanding^30^. To fully realize this potential, we need to be cognizant that the promise here is not about the hype around AI capabilities today^31^, but that robust infrastructures are essential to facilitate search, computation, visualization, and collaboration, as well as improvement of these AI systems. Infrastructural ecosystems are critical for accelerating AI for neuroscience and creating synergy across institutions for knowledge discovery, validation, translation, and clinical applications. In the next section I discuss eight challenges that are limiting the transition to a more effective ecosystem for science.

## Challenges in the Current Neuroscience Ecosystem

### 1. Complexity of Neuroscientific Inquiry

Neuroscientific inquiry has evolved into a remarkably rich and complex endeavor, encompassing a vast array of sophisticated methodologies and investigative approaches, spanning diverse domains. For example, one can capture in vivo brain activity using technologies like Neuropixels probes that enable the simultaneous recording from hundreds of neurons in awake behaving animals^32^ (including humans^33^). As another example, ex vivo examination of human brain tissue can involve advanced tissue-clearing and expansion microscopy techniques^34,35^. These render samples transparent and/or physically enlarged to enable unprecedented observation of gene expression and proteins at cellular and sub-cellular resolution^35^. Such detailed insights are essential for detecting subtle, yet significant, differences between individuals, especially when studying neurodegenerative conditions such as Alzheimer’s Disease and Related Dementias (ADRD).

Each neuroscientific experiment, regardless of its specific focus, demands a highly detailed and multistep protocol, integrating a multitude of advanced technologies. Executing such workflows requires meticulous planning, rigorous implementation, and seamless integration of diverse technological platforms. In many situations these new questions require new instruments, new algorithms to analyze the new data, and new models of the system under study. This makes neuroscience today inherently interdisciplinary: progress depends not only on deep biological expertise but also on mastery of engineering, data science, software development, cloud operations, and increasingly, AI.

### 2. The Data Explosion

As already touched on, a key challenge is the volume of data. As the neuroscience community pushes the boundaries of what can be observed and quantified, the volume of data generated has surged exponentially. Neuroscience has moved from collecting megabytes (MB) and gigabytes (GB) of data to routinely handling terabytes (TB) and increasingly, petabytes (PB). This scale is driven by the complexity of experiments, the resolution of modern instruments, and the ambition to capture multi-species, multi-scale phenomena across time. Such a dramatic increase in data volume presents both unprecedented opportunities and serious challenges^36^. It necessitates worldwide infrastructures that are not just storage solutions, but end-to-end platforms enabling data curation, provenance tracking, cross-site collaboration, computation, and long-term access.

Many repositories are distributing PBs of data around the world resulting in significant new knowledge from existing datasets, reducing some of the needs for new data collection and its associated costs and resource consumption. However, even knowing what data exists is a challenge, and most primary data are not making their way out of labs in a timely manner, with many PIs and institutions holding on to data that has potential beyond the reasons for which they were generated. Thus, there is an incomplete picture of the data that exists in the world at any point in time, and no way to evaluate whether such data can answer pressing questions. There is also no effort to collectively influence and improve the type and quality of data to be collected.

No infrastructure exists to coordinate large scale data collection and integration. A system capable of indexing, accessing, and assessing data at any instant could address many of these challenges. It would also immediately serve the needs for training and validating AI models.

### 3. Fragmentation and Lack of Coordination

The lack of coordination of common standards or a “lingua franca” for data formats, metadata, analysis pipelines, and sharing protocols severely impedes collaboration and data reuse and increases potential for errors and miscommunication. As a result, there is great variation in the quality and precision of information across many resources being generated and used. Such imprecision, in turn, influences the reliable training of AI models and the correctness of AI systems.

Despite several advances to consolidate practices across some communities in neuroscience, overall it remains largely a “cottage industry” in which individual labs often develop bespoke protocols and software solutions for specific, localized problems. While this independence fosters creativity and expediency, it undermines interoperability. Data generated in one lab may be incompatible with tools used in another, rendering integration or consolidation difficult or impossible. Such one-off solutions also increase the potential risk for errors and sustainability when compared to community-maintained, well-engineered software systems.

Standards such as BIDS (Brain Imaging Data Structure^37^), NWB (Neurodata Without Borders^38^), and OME-Zarr^39^ have made significant strides in community-driven harmonization of data formats and metadata across domains like neuroimaging, neurophysiology, and microscopy. However, adoption remains inconsistent despite being driven by public data repositories. Incentives for using such standards are limited, and many researchers face steep learning curves or lack institutional support to transition. Further, a lack of institutional or other resources and priorities reduces engagement with the development of these standards, leading to significant lags in the timely deployment and utility of these resources. The efforts of the International Neuroinformatics Coordinating Facility (INCF^40^), an international body originally established as an outcome of Organisation for Economic Co-operation and Development (OECD), delivered early and coordinated progress in neuroinformatics, but it has since been severely limited in its efforts as a result of policy changes and funding priorities across nations. We are more fragmented today as a result of this reduced coordination.

### 4. Skill and Knowledge Gaps Persist Across the Ecosystem

Infrastructure is only as useful as the ability of researchers to effectively utilize it. The use of complex data platforms, computational workflows, and AI models requires specialized expertise in software engineering, data management, and statistical and algorithmic reasoning. However, access to training and technical support is highly uneven. Many labs lack personnel with the necessary computational background, and even where training is available, it may not be tailored to the specific needs of neuroscience. This expertise gap limits the reuse of data, the development of robust pipelines, and the reproducibility of results. Moreover, it creates a dependence on a small number of highly skilled individuals, leading to bottlenecks in collaborative projects and contributing to burnout and attrition.

While the complexity of neuroscience has increased, federal support for training programs has reduced, especially in the US. This has reduced broad baseline knowledge and skills that can contribute to career stability and resilience. As AI systems are increasingly used, fundamental questions arise around the role of education and the future of work. Training itself needs to reflect these changing directions, but is not adapting quickly to the current nature of technological changes. Many individuals are turning to AI systems for education, without a clear consideration of the ability or inability of these systems to deliver accurate knowledge. Thus, there is a significant potential for an even more brittle workforce and scientific ecosystem.

### 5. Infrastructure Inequities

One of the most pressing issues is the striking variability in infrastructural resources across labs, institutions, and countries. In contrast to those in under-resourced settings, researchers in well-funded institutions in high-income countries often enjoy access to state-of-the-art equipment, dedicated IT support, scalable storage, and high-bandwidth networks. This imbalance limits participation in large-scale collaborative science and introduces systemic challenges in who can contribute to and benefit from cutting-edge research. It also poses significant obstacles to building globally representative datasets. Even worse, as those with limited resources and varied expertise create, as they must, ad hoc, limited-scope, overly specific systems, their efforts further imbalance the system, and perpetuate a cycle of dependence on increasing funding simply to maintain these systems.

### 6. Ethical and Regulatory Fragmentation

In addition to technical and resource disparities, neuroscientific infrastructure must navigate complex ethical and regulatory landscapes. Data privacy, security, and consent, particularly for human data, are governed by diverse frameworks and policies that vary by country and institution. These discrepancies can complicate international collaborations, hinder data sharing, and delay scientific progress. Given some of the health-related challenges neuroscientists are trying to solve, the collection and exchange of information across the world’s population is an essential requirement for the development of robust treatments. We have already seen inconsistencies in diagnosis of mental health disorders and selection of treatments on a trial and error basis. As AI systems begin to interact more deeply with neuroscientific datasets in the march towards precision medicine, these ethical concerns will only grow. AI models trained on poorly governed data may perpetuate biases or expose sensitive information.

### 7. Biased Attribution

Neuroscience is a team science. Every advance stems from the dedication and contributions of many individuals. However, in the contemporary landscape of academia and industry, the relentless pursuit of limited tenure-track positions, grant funding, and competitive industry roles has fostered an environment where quantity often overshadows quality. Individual credit is derived through publications. The “publish or perish” mentality incentivizes researchers to produce a high volume of papers, sometimes leading to fragmented research, minimal new insights, or rushed submissions. The pressure to publish often means that the development of robust, well-documented, and easily usable software takes a backseat. The individuals and teams responsible for building and sustaining infrastructural and software elements often receive inadequate recognition^41^, leading to lack of maintenance of these elements and hindering reproducibility. Such trends highlight a systemic issue where the current reward structures often fail to adequately recognize the diverse forms of contribution that are essential for scientific advancement.

### 8. Infrastructure is Underfunded

The foundational infrastructure crucial for scientific progress, particularly in fields like neuroscience, is severely underfunded, creating a severe bottleneck for innovation and scalability. A portion of infrastructure is usually supported through indirect costs associated with institutional components. However, a vast and increasingly critical segment of neuroscience infrastructure - encompassing data coordinating and dissemination centers, sophisticated data repositories, and the essential work of software development and engineering^42^ does not receive funding commensurate with the escalating needs of scientific projects. This disproportionate funding model prevents these vital components from scaling effectively^43^ to meet the demands of modern research. While proposals to address this disparity have emerged, notably within the European Union, they have yet to translate into concrete policy changes and sustained funding mechanisms, which are themselves under attack^44^. The development and deployment of sophisticated AI models demand substantial investments in computational power, extensive data storage, and specialized engineering expertise. Both funds and expertise are increasingly moving to large technology companies, who do not see commercial return of investment in fundamental research, and who do not fund such research. Without a significant recalibration of funding priorities, the burgeoning potential of AI in scientific discovery will be severely hampered by inadequate infrastructure, widening the gap between scientific ambition and practical execution.

## Recommendations for an AI-Aligned Neuroscience Infrastructure

The challenges in the previous section are all interconnected, and yet we are still tackling them piecemeal, thus failing to resolve them. The neuroscientific community should shed these piecemeal efforts and collectively re-engineer the entire infrastructure that can support a more seamless and collaborative scientific process. Over the past two decades, my work in neuroscience has involved leading numerous initiatives to develop standards, software, and infrastructure. This extensive experience has provided me with ample opportunities to observe, reflect on, and evaluate the impact of these efforts, and to interact with many researchers throughout the process.

In many of these interactions, the FAIR^45^, CARE^46^, and TRUST^47^ principles (see Box 1) frequently emerge as invaluable. The FAIR (Findable, Accessible, Interoperable, and Reusable) principles for data are widely recognized as the global standard for effective data management. These are complemented by the CARE (Collective benefit, Authority to control, Responsibility to providers, and Ethics) principles for Indigenous Peoples’ data governance (I must make an extra note here that while the CARE principles were developed with Indigenous Peoples’ rights and interests in mind, I use them more broadly as guidelines that should serve all digital object governance). More recently, the TRUST (Transparency, Responsibility to users, User focus, Sustainability, and Technology) principles were developed for improving digital repositories. But, whereas these principles establish an important foundation for scientific values that broadly apply to any digital infrastructure, their operationalization remains severely haphazard. The varied approaches to implementing these principles have, in fact, led to many of the challenges highlighted previously.

Based on my observations and interactions, and building on values encoded in the FAIR, CARE, and TRUST principles, I outline operational recommendations to guide the transformation towards a more dynamic, self-learning, and self-correcting ecosystem in which to do our scientific work. If adopted, these should allow an organic growth of connected, decentralized, and robust infrastructure that can self-adjust and self-organize over time adapting to the needs of a scientific community.

### Build Modular, Self-Describing Components

To foster broad participation in scientific development, our future infrastructure should be modular, pluggable, and fully sharable. Each module, whether a data pipeline, AI model, instrument, or service, requires clearly defined and described inputs, operations, and outputs. Modules must be self-describing, answering basic questions about their function and allowing for validation of their functionality. This design promotes cohesion and agility, accelerated experimentation, scalability, and obviates the need to re-architect systems from scratch. To avoid confusion, inefficiency, and resource wastage from uncoordinated, redundant, or overlapping modules, systems should analyze the downstream impact of updates and identify underused or outdated modules for deprecation. Governance must enable broad participation in module addition while upholding strict quality and integration standards for long-term resilience.

Modules should also avoid tight coupling, which can lead to unpredictable cascading changes or an inability to accommodate evolving standards, resulting in degraded performance or loss of interoperability. The aim is to ensure robustness without compromising stability, allowing for rapid iteration while maintaining a stable yet evolving system. Each module will function as a computational unit capable of integration into both human and machine feedback loops, and every input and output will be a self-describing packet of information.

### Embed Fast Feedback Loops

A feedback-responsive system is fundamental to scientific infrastructure, as it supports short feedback cycles among data collection, analysis, interpretation, and system refinement, ensuring agility and responsiveness to emerging needs and discoveries. In software development, current infrastructures close the loop by linking user feedback to issues, issues to changes to software, and changes to automated evaluations that confirm the fix. Much like this, a scientific system should produce signals that enable actions through feedback loops, whether by humans or machines.

To operationalize this, feedback must be embedded into scientific workflows at all levels, allowing infrastructure to constantly improve, correct errors, and refine models, mirroring the scientific method itself. The time from feedback to response should be continuously monitored and optimized for reduction throughout the system. This approach addresses the risk of issues persisting unnoticed, leading to stagnation or reduced trust, by tracking the time between issue identification and resolution, analyzing user feedback channels for recurring themes, monitoring how frequently updates and improvements are made, and assessing whether system-level changes align with observed usage patterns.

### Adopt Dynamic Schemas With Semantics

For scientific infrastructure to participate as an active contributor to scientific work, it is fundamental that systems make data, tools, and processes understandable to both humans and machines through well-defined schemas, ontologies, and terminologies. The goal of improving shared understanding should be built into the design and operation of future systems. Structured and semantic representations enable AI systems to interpret, reason, and act meaningfully across domains, while also supporting humans in evaluating and interpreting outputs.

To operationalize this, we must ensure dynamic schemas that can incorporate new concepts as easily as exposing and refining older concepts as our understanding of the space changes. If every data element in this ecosystem was structured with a machine-readable description and its provenance^48^, it would solve most of the common challenges that are currently encountered in schema development, and would enable dynamic schemas and seamless data movement. Any prior information should be checked for validity against new schemas, identifying elements invalidated by the new or refined scientific models. This proactive approach tackles the risks of opaque and difficult-to-interpret data when clear, shared definitions are lacking or evolve without synchronization. It directly counters schema drift, where ontologies and models evolve inconsistently, preventing broken interoperability and confusion, particularly for AI systems that rely on semantic precision. Furthermore, by making metadata addition intuitive and integrated, we mitigate the risk of sparse, ambiguous, or manually intensive metadata that researchers might bypass, leading to low-quality annotations and fragmented datasets.

### Continuously Prune & Adapt (Self-Healing Ops)

For scientific infrastructure to remain relevant and trustworthy, it is fundamental that systems regularly review, correct, and prune outdated, low-quality, or redundant components (instruments, data, algorithms, knowledge), while simultaneously evolving with new data and techniques. This dynamic adaptation is critical for AI systems that depend on high-quality inputs and must be able to adapt to new contexts and knowledge without excessive human micromanagement.

To operationalize this, a monitoring system must be embedded within the infrastructure to proactively address decay. Furthermore, systems should incorporate automated quality (re)evaluations, use automated tests to flag failures, and support streamlined processes to archive, update, or deprecate components when they are no longer fit for purpose. This proactive approach directly counters the risks of stagnant, brittle, and irrelevant systems that accumulate technical debt, consuming resources and diminishing trust.

### Manage Resource Lifecycles (Persistent vs. Transient)

For scientific infrastructure to optimize resource utilization and maintain clear governance, it is fundamental to differentiate between persistent and transient resources. Transience allows for rapid experimentation, while persistence ensures reproducibility and continuity. This is crucial for intelligent systems, as AI models rely on well-defined data lineage and stable core interfaces to function effectively.

To operationalize this, systems must implement mechanisms to tag artifacts with their intended lifespans. This involves auditing archival completeness to ensure critical data are preserved, while simultaneously implementing automated expiration or cleanup policies for temporary data. Furthermore, active monitoring of user expectations regarding data availability over time will help prevent accidental deletion of needed transient data or the cluttering of storage by ephemeral outputs that persist beyond their usefulness.

### Enforce End-to-End Provenance & Audits

Scientific trust is severely undermined when the origins and transformations of data and models are undocumented or lost, leading to irreproducible results, misattribution, or undetected misuse. Every data element in a dataset, model, and decision must be fully traceable from its origin to its current state. This fundamental need is addressed by robust provenance and auditability, a transparent historical record of creation, evolution, and trustworthiness. This is especially critical for intelligent systems, as audit trails enable reproducibility and validation, fostering trust in AI outputs and allowing systems to self-diagnose or explain their reasoning. For humans, it facilitates crucial evaluation and correction, and perhaps more importantly attribution.

To operationalize this, a continuous verification system is essential, ensuring that lineage is preserved from data acquisition through to analysis and publication. This involves consistently checking for complete provenance metadata, validating the integrity of data and model histories, ensuring that decisions made by AI models are explainable, and rigorously auditing access and change logs for transparency and accountability.

### Co-Design Usable, Inclusive Human-AI Systems

It is fundamental that systems effectively serve the diverse needs of real people, individuals with varying backgrounds, expertise, and goals. We must create intuitive, inclusive designs for all users, extending beyond technical experts to encompass the entire scientific community. To operationalize this user-centered approach, we must implement intuitive user interfaces and workflows, and improve user experience through enabling AI-driven interactions. All of this should be co-designed with the users with diverse skills, backgrounds, and abilities. This should be a continuous process driven by the user and their use-cases. New developments in technologies now make it possible for us to define, design, and deliver such a dynamic process, capable of resolving many issues simply through interactions with autonomous AI agents.

### Make All Digital Components Accessible Programmatically

For scientific infrastructure to truly empower advanced AI collaboration and ensure robust reproducibility, scientific work must be encoded in ways that are not only human-readable but also machine-actionable. The solution lies in making data, methods, protocols, results, and decisions accessible programmatically. This fundamental shift is critical because, to integrate AI as a scientific collaborator, the system must be fully programmable, capable of simulating hypotheses, running experiments, adapting to new knowledge, and learning autonomously, thereby enabling self-adjusting, AI-augmented science.

To operationalize this, systems must actively track how much of the infrastructure is scriptable and testable, providing a clear metric of programmability. Furthermore, rigorous measurement of reproducibility rates across analyses is essential to ensure that computational workflows can be consistently rerun and validated independently, preventing results from becoming untrustworthy. Actively monitoring the health and usage of programmatic interfaces will prevent them from becoming inadequate or inconsistent, while ensuring that computational workflows are machine-readable and properly versioned directly counters the risk of missing execution metadata.

### Activate Rapid, AI-Assisted Coordination

For scientific infrastructure to address complex scenarios effectively, it is fundamental to overcome fragmented knowledge and foster efficient problem-solving. The absence of readily available, relevant expertise often leads to prolonged decision-making, duplicated efforts, and suboptimal outcomes. Ensuring rapid coordination addresses this by leveraging collective intelligence and AI-driven knowledge integration to provide immediate access to critical insights.

To operationalize this, we must coordinate all components of this infrastructure. This includes having a coordination group, establishing platforms for identifying and engaging subject matter experts across diverse fields, facilitating real-time collaboration, and knowledge sharing. AI systems should be integrated to capture and organize distributed knowledge including mistakes, null findings, and practical solutions, making it searchable and easily retrievable. Collaborative tools must exist that facilitate identifying commonalities and divergences in expert opinions (e.g. platforms like Polis^49^). This proactive approach directly counters the risks of inefficiency and missed opportunities that arise from disconnected knowledge. It ensures efficient decision-making and fosters innovation by integrating diverse perspectives.

## Conclusion: A Call For Change

Infrastructure is not a static scaffold around science. It is a dynamic system that can either accelerate or impede discovery. Neuroscience, with its multimodal and multiscale data, intricate methods that probe multiple species, and profound and urgent societal implications, is both the stress test and the proving ground for a new model: an intelligent, AI-aligned ecosystem that learns from use, coordinates efforts across communities, and continually improves itself.

The recommendations outlined here are requirements for an infrastructure of the future in which humans and AI collaborate productively. They operationalize FAIR, CARE, and TRUST principles by turning values into implementation guidelines: interfaces we can depend on, metadata we can compute with, decision trails we can audit, and systems that adapt with agility as questions change. The costs of staying with today’s fragmented, brittle infrastructure are already visible. We waste energy, both computation and human effort, by duplicating tools and infrastructure and regenerating and curating data that should have been FAIR from the start. We open channels for misinformation and disinformation when provenance is weak, incentives reward volume over verification, and many scientific results cannot be reproduced or audited. We widen inequities when only a few institutions can afford to participate at scale, and we drain talent when software and data work remain under-recognized and underfunded. These are not abstract risks, they are daily inhibitors of scientific progress.

I suspect that many of my colleagues agree with the challenges and potential solutions proposed here and the need for tackling them holistically. But, almost none are in a position to enact this scale of simultaneous change, and many are not in a position to augment their current research portfolios to take on these challenges. Some would argue we need evidence of impact before scaling, we need to carry out efforts aligned with our limited resources, and perhaps neuroscience does not need such a globally coordinated infrastructure. To them I say that we are limited in the questions we can ask, and the limits of our answers, because of the lack of such a coordinated infrastructure, and that a practical path forward is within reach.

Funders can create long-horizon, renewable infrastructure lines, treating data coordination centers, software engineering, and compute commons as core scientific instrumentation, and perhaps most importantly providing support for coordination across global efforts. Institutions and journals can require machine-actionable data and metadata, registered analysis pipelines, and reproducible computation environments, while crediting software, data curation, and infrastructure leadership as first-class scholarly outputs. Consortia can converge on shared interfaces and ontologies, with governance that allows rapid evolution as needs change. Training programs can close skill gaps by pairing domain science with engineering and AI literacy^50^, and by valuing negative results and careful curation. And globally, we can lower barriers for under-resourced communities by investing in shared platforms, harmonized ethics frameworks, and equitable access to compute and storage. This increased representation is critical for an AI-enabled ecosystem.

AI’s role in this ecosystem will be critical. AI systems can help ascertain what we know across scientific domains and how precisely we know it and help design scalable experiments and systems for the most complex questions. They will also monitor data as it emerges, orchestrate collaborative analyses, and propose next steps towards society’s biggest challenges. But, this will only happen if the substrate is programmable, provenance-rich, and auditable. With guardrails in place, AI can make science faster and more reliable while preserving human judgment where it matters most: setting goals, weighing risks, and interpreting meaning.

The destination is an infrastructure that fades into the background because it simply works, like our nervous, circulatory, and immune systems, quietly coordinating signals, routing resources, detecting anomalies, and healing itself when something goes wrong. Building it will require a conscious and deliberative effort towards collective design, sustained investment, and new reward structures. The payoff is a science enterprise that is more rigorous, more inclusive, and dramatically more capable of turning data into understanding and understanding into better lives. If we treat intelligent infrastructure as a central instrument of discovery, not an afterthought, we can meet the complexity of brain science head-on and establish a blueprint other fields can adopt.

## Figures and Tables

**Figure 2.**
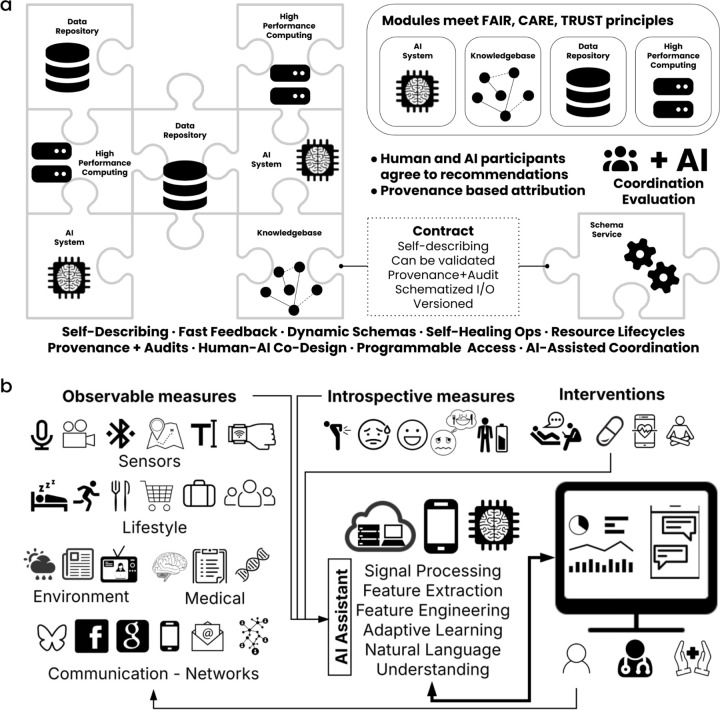
**a,** The proposed infrastructural fabric will allow plugging in a wide assortment of modules that operationalize the FAIR, CARE, and TRUST principles. This community governed and coordinated setup will allow human and AI participation as long as each module or service agrees to a contract to implement the guidelines. **b,** A use-case illustrating how the implementation of the recommendations will enable all aspects necessary for data, AI analytics, clinical and individual participation to co-exist and scale, allowing more effective research towards precision psychiatry.

## Box

**Box 1.** The FAIR, CARE, and TRUST principles. These principles provide a foundation of values that should apply to any infrastructure, especially an AI-embedded one. As much as these principles define guidelines for data management, Indigenous Peoples’ data governance, and digital repositories, the recommendations in this perspective help operationalize these guidelines towards implementation in an intelligent infrastructure for science.

Principle	Primary focus	Who it targets	Relation to perspective
**FAIR** **F**indable **A**ccessible **I**nteroperable **R**eusable	“ability of machines to automatically find and use the data, in addition to supporting its reuse by individuals.”	Data producers & users (human + machine)	All components of infrastructure should operate under FAIR considerations.
**CARE** **C**ollective Benefit **A**uthority to Control **R**esponsibility **E**thics	“encouraging open and other data movements to consider both people and purpose in their advocacy and pursuits.”	Indigenous Peoples and communities and their data users	All components of an infrastructure should adhere to these CARE principles including AI systems, paying attention to data sources.
**TRUST** **T**ransparency **R**esponsibility **U**ser focus **S**ustainability **T**echnology	“remind data repository stakeholders of the need to develop and maintain the infrastructure to foster continuing stewardship of data and enable future use of their data holdings”	Repository operators & their users	All components of an infrastructure need to provide TRUST through communal governance and stewardship.

Sources: FAIR: https://www.nature.com/articles/sdata201618, CARE: https://www.gida-global.org/care, TRUST: https://www.nature.com/articles/s41597-020-0486-7

## References

[1] van Laak D. Lifelines of Our Society. (MIT Press, Cambridge, MA, USA, 2023).

[2] Güntsch A. National biodiversity data infrastructures: ten essential functions for science, policy, and practice. Bioscience 75, 139–151 (2025). 40060162 10.1093/biosci/biae109 PMC11884780

[3] UNESCO. UNESCO Recommendation on Open Science. https://doi.org/10.54677/MNMH8546 (2021) doi:10.54677/mnmh8546.

[4] Azevedo F. A. C. Equal numbers of neuronal and nonneuronal cells make the human brain an isometrically scaled-up primate brain. J. Comp. Neurol. 513, 532–541 (2009). 19226510 10.1002/cne.21974

[5] Ngai J. BRAIN @ 10: A decade of innovation. Neuron 112, 3003–3006 (2024). 39326390 10.1016/j.neuron.2024.09.007 PMC11502121

[6] Iyer S. The BRAIN Initiative data-sharing ecosystem: Characteristics, challenges, benefits, and opportunities. Elife 13, (2024). 10.7554/eLife.94000 PMC11602185 39602224

[7] Amunts K. BigBrain: an ultrahigh-resolution 3D human brain model. Science 340, 1472–1475 (2013). 23788795 10.1126/science.1235381

[8] Dorkenwald S. Neuronal wiring diagram of an adult brain. Nature 634, 124–138 (2024). 39358518 10.1038/s41586-024-07558-y PMC11446842

[9] A state-of-the-art ecosystem for neuroscience. EBRAINS https://www.ebrains.eu/about.

[10] Casey B. J. The Adolescent Brain Cognitive Development (ABCD) study: Imaging acquisition across 21 sites. Dev. Cogn. Neurosci. 32, 43–54 (2018). 29567376 10.1016/j.dcn.2018.03.001 PMC5999559

[11] Di Martino A. The autism brain imaging data exchange: towards a large-scale evaluation of the intrinsic brain architecture in autism. Mol. Psychiatry 19, 659–667 (2014). 23774715 10.1038/mp.2013.78 PMC4162310

[12] Markiewicz C. J. The OpenNeuro resource for sharing of neuroscience data. Elife 10, (2021). 10.7554/eLife.71774 PMC8550750 34658334

[13] Van Essen D. C. The WU-Minn Human Connectome Project: an overview. Neuroimage 80, 62–79 (2013). 23684880 10.1016/j.neuroimage.2013.05.041 PMC3724347

[14] Thompson P. M. The ENIGMA Consortium: large-scale collaborative analyses of neuroimaging and genetic data. Brain Imaging Behav. 8, 153–182 (2014). 24399358 10.1007/s11682-013-9269-5 PMC4008818

[15] Weiner M. W. The Alzheimer’s Disease Neuroimaging Initiative: a review of papers published since its inception. Alzheimers. Dement. 9, e111–94 (2013). 23932184 10.1016/j.jalz.2013.05.1769 PMC4108198

[16] Olén O. & H Everhov Å. An updated review of The Swedish National Patient Register as a data source. Lakartidningen 122, (2025). 40776807

[17] Everhov Å. H. Diagnostic accuracy in the Swedish national patient register: a review including diagnoses in the outpatient register. Eur. J. Epidemiol. 40, 359–369 (2025). 40140143 10.1007/s10654-025-01221-0 PMC12137447

[18] Bycroft C. The UK Biobank resource with deep phenotyping and genomic data. Nature 562, 203–209 (2018). 30305743 10.1038/s41586-018-0579-z PMC6786975

[19] Moor M. Foundation models for generalist medical artificial intelligence. Nature 616, 259–265 (2023). 37045921 10.1038/s41586-023-05881-4

[20] Hosseini M., Horbach S. P. J. M., Holmes K. & Ross-Hellauer T. Open Science at the generative AI turn: An exploratory analysis of challenges and opportunities. Quant. Sci. Stud. 6, 22–45 (2025). 40124128 10.1162/qss_a_00337 PMC11928019

[21] Liu J. Neural Brain: A neuroscience-inspired framework for embodied agents. arXiv [cs.RO] (2025).

[22] Caro J. O. BrainLM: A foundation model for brain activity recordings. bioRxiv (2023) doi:10.1101/2023.09.12.557460.

[23] Luo X. Large language models surpass human experts in predicting neuroscience results. Nat. Hum. Behav. 9, 305–315 (2025). 39604572 10.1038/s41562-024-02046-9 PMC11860209

[24] Zeng Y. CellFM: a large-scale foundation model pre-trained on transcriptomics of 100 million human cells. Nat. Commun. 16, 4679 (2025). 40393991 10.1038/s41467-025-59926-5 PMC12092794

[25] Talukder S., Yue Y. & Gkioxari G. TOTEM: TOkenized Time Series EMbeddings for general time series analysis. arXiv [cs.LG] (2024).

[26] Guo F. Foundation models in bioinformatics. Natl. Sci. Rev. 12, nwaf028 (2025). 40078374 10.1093/nsr/nwaf028 PMC11900445

[27] Schrimpf M. Integrative Benchmarking to Advance Neurally Mechanistic Models of Human Intelligence. Neuron 108, 413–423 (2020). 32918861 10.1016/j.neuron.2020.07.040

[28] Kell A. J. E., Yamins D. L. K., Shook E. N., Norman-Haignere S. V. & McDermott J. H. A Task-Optimized Neural Network Replicates Human Auditory Behavior, Predicts Brain Responses, and Reveals a Cortical Processing Hierarchy. Neuron 98, 630–644.e16 (2018). 29681533 10.1016/j.neuron.2018.03.044

[29] Mahmood U., Fu Z., Ghosh S., Calhoun V. & Plis S. Through the looking glass: Deep interpretable dynamic directed connectivity in resting fMRI. Neuroimage 264, 119737 (2022). 36356823 10.1016/j.neuroimage.2022.119737 PMC9844250

[30] Johnson E. C. SciOps: Achieving productivity and reliability in data-intensive research. arXiv [q-bio.NC] (2023).

[31] Narayanan A. & Kapoor S. AI Snake Oil: What Artificial Intelligence Can Do, What It Can’t, and How to Tell the Difference. (Princeton University Press, Princeton, NJ, 2024).

[32] International Brain Laboratory. Electronic address: churchland@cshl.edu & International Brain Laboratory. An international laboratory for systems and computational neuroscience. Neuron 96, 1213–1218 (2017). 29268092 10.1016/j.neuron.2017.12.013 PMC5752703

[33] Paulk A. C. Large-scale neural recordings with single neuron resolution using Neuropixels probes in human cortex. Nat. Neurosci. 25, 252–263 (2022). 35102333 10.1038/s41593-021-00997-0

[34] Glaser A. Expansion-assisted selective plane illumination microscopy for nanoscale imaging of centimeter-scale tissues. (2025) doi:10.7554/elife.91979.3. PMC12208669 40586775

[35] Park J. Integrated platform for multiscale molecular imaging and phenotyping of the human brain. Science 384, eadh9979 (2024). 38870291 10.1126/science.adh9979 PMC11830150

[36] Schottdorf M., Yu G. & Walker E. Y. Data science and its future in large neuroscience collaborations. Neuron 112, 3007–3012 (2024). 39326391 10.1016/j.neuron.2024.08.017 PMC11632705

[37] Gorgolewski K. J. The brain imaging data structure, a format for organizing and describing outputs of neuroimaging experiments. Sci Data 3, 160044 (2016). 27326542 10.1038/sdata.2016.44 PMC4978148

[38] Rübel O. The Neurodata Without Borders ecosystem for neurophysiological data science. Elife 11, e78362 (2022). 36193886 10.7554/eLife.78362 PMC9531949

[39] Moore J. OME-Zarr: a cloud-optimized bioimaging file format with international community support. Histochem. Cell Biol. 160, 223–251 (2023). 37428210 10.1007/s00418-023-02209-1 PMC10492740

[40] Abrams M. B. A standards organization for open and FAIR neuroscience: The International Neuroinformatics Coordinating Facility. Neuroinformatics 20, 25–36 (2022). 33506383 10.1007/s12021-020-09509-0 PMC9036053

[41] Westner B. U. Cycling on the Freeway: The perilous state of open-source neuroscience software. Imaging Neurosci. (Camb.) 3, (2025). 10.1162/imag_a_00554 PMC12319967 40800958

[42] Katz D. S., Jensen E. A. & Barker M. Understanding and advancing research software grant funding models. Open Res. Eur. 5, 199 (2025). 10.12688/openreseurope.20210.2 PMC12743209 41458369

[43] List J. A. Optimally generate policy-based evidence before scaling. Nature 626, 491–499 (2024). 38356064 10.1038/s41586-023-06972-y

[44] Jalali M. S. & Hasgul Z. Potential trade-offs of proposed cuts to the US National Institutes of health. JAMA Health Forum 6, e252228 (2025). 40711777 10.1001/jamahealthforum.2025.2228

[45] Wilkinson M. D. The FAIR Guiding Principles for scientific data management and stewardship. Sci Data 3, 160018 (2016). 26978244 10.1038/sdata.2016.18 PMC4792175

[46] Carroll S. R. The CARE Principles for Indigenous Data Governance. CODATA 19, 43–43 (2020).

[47] Lin D. The TRUST Principles for digital repositories. Sci. Data 7, 144 (2020). 32409645 10.1038/s41597-020-0486-7 PMC7224370

[48] Moreau L. & Missier P. PROV-DM: The PROV Data Model. (World Wide Web Consortium, 2013).

[49] The Computational Democracy Project. https://compdemocracy.org/.

[50] Better Code, Better Science — Better Code, Better Science. https://poldrack.github.io/BetterCodeBetterScience/frontmatter.html.

